# Supplementary material for: ‘Misdiagnosed and Misunderstood’: Insights into Rarer Forms of Dementia through a Stepwise Approach to Co-Constructed Research Poetry
Source: Healthcare (Basel). 2024 Feb 17;12(4):485. doi: 10.3390/healthcare12040485 (PMC10887667; doi:10.3390/healthcare12040485)
Supplement: Supplementary file 1 [file healthcare-12-00485-s001.zip › healthcare-2753389-supplementary/Figure-S1.pdf]

Supplemental Figure S1: Dementia Types and Characteristics

Type of Dementia	Main Characteristics
Familial Alzheimer's Disease (FAD)	A genetically inherited form of Alzheimer's disease. Most cases of FAD are caused by mutations in the genes presenilin 1 (PSEN1), presenilin 2 (PSEN2) and amyloid precursor protein (APP). The symptoms of FAD are usually similar to typical Alzheimer's disease (i.e. primarily memory loss). People with FAD most commonly first develop symptoms in their 40s or 50s but this varies considerably between families. Some individuals may be affected as early as in their 30s [23].
Behavioural Variant Frontotemporal Dementia (bvFTD)	A form of dementia characterised by personality and behaviour changes including apathy, and a progressive decline in socially appropriate behaviour, judgement, self-control, and empathy. Memory is usually relatively well-preserved in bvFTD but people with bvFTD may lack insight into their symptoms and the impact they have on others [24].
Primary Progressive Aphasia (PPA)	The Primary Progressive Aphasias (PPAs) are a group of dementias mainly characterised by progressive problems with speech and language [25]. Three subtypes of PPA are:<br>1. Nonfluent Variant Primary Progressive Aphasia (nfvPPA): Early symptoms of agrammatism, apraxia of speech and phonemic errors<br>2. Semantic dementia (SD): Impaired comprehension and ability to match words with images or meanings<br>3. Logopenic progressive aphasia (LPA): Impaired single-word retrieval, naming and sentence repetition
Posterior Cortical Atrophy (PCA)	PCA is a visually-led form of dementia characterised by progressive decline in visual processing skills, and relatively intact memory and language in the early stages [26]. Common first signs and symptoms include difficulties with seeing what and where things are (for example, when driving or reading). These include problems with recognition, difficulty with coordination, issues with judging distances, light sensitivity and difficulty interpreting, locating, or reaching for objects.
Lewy Body Dementia (LBD)	An umbrella term that includes dementia with Lewy Bodies and Parkinson's disease dementia. LBD can affect thinking and memory, and also cause visual hallucinations (seeing things that are not there), as well as movement changes similar to Parkinson's disease [27].

Young-Onset Alzheimer's Disease (YOAD)	About 5% of people with Alzheimer's disease are under 65. This is called early-onset or young-onset Alzheimer's, usually affecting people in their 40s, 50s and early 60s [5]. Symptoms may include other impairments where memory loss is not the main symptom (e.g., language impairment, vision difficulties [28].
