# Supplementary material for: ‘Misdiagnosed and Misunderstood’: Insights into Rarer Forms of Dementia through a Stepwise Approach to Co-Constructed Research Poetry
Source: Healthcare (Basel). 2024 Feb 17;12(4):485. doi: 10.3390/healthcare12040485 (PMC10887667; doi:10.3390/healthcare12040485)
Supplement: Supplementary file 1 [file healthcare-12-00485-s001.zip › healthcare-2753389-supplementary/Figure-S2.pdf]

Supplemental Figure S2: Data Set including poems, source material and explanations of the poets' creative process

Rare Dementia Support Impact Study

This data set contains 27 completed poems from 71 participants (9 cohort groups), explanations of the poets' creative process, and source material (original words) from people living with rare dementia and carers (stakeholders) who responded to a series of three prompts over a 12 week period. Data was collected between 2021-2022 as part of the Electronic Poems Project within the Rare Dementia Support Impact Study. Survey data from healthcare professionals and students (collected between 2022-2023) is available from the corresponding author with appropriate justification for its use.

The data are organised by: Cohort group (numbered 5-13), Prompt number (1-3)/*Prompt question.* For example, immediately below, 5 refers to cohort 5 and 1 refers to prompt 1 (out of 3 prompts); 5.2 refers to cohort 1 and prompt 2, whereas 5.3 refers to cohort 5 and prompt 3. The prompts are in italics. This is followed by the poem, the poet's explanation and source material. The structure of each poem is reproduced in the same format it was sent to participants.

5.1/*What is your experience of having PCA or being a family member caring for someone with PCA?*

A Steep Learning Curve

A steep learning curve

Always on duty

Anxiety oh dear oh dear

    Always

Challenging symptoms and behaviours

Connect with the inner spirit beyond the inabilities

    Connect

Deep compassion

Exhaustion leading to chronic fatigue

Deep

Feeling of worthlessness

Feeling restricted

Frustration

Frustration navigating the health care and social care system

Feeling

I remember being mindful with my breathing as I consciously joined them in their reality

Independence lost

Remember

Lost for words

Misunderstood and fragile

Needing to cope with boredom

Words

Often there was silence in the room although their energy (a different kind of energy to when they were well) was very much still there

Silence

Remembering happier times

Stuck in a loop, repeating the same actions everyday

Time slows down and remembering to truly be in the moment

Time

Unable to make them well again

Unfinished business, unable to complete or explain

Unfinished

A steep learning curve

Learning

Poet's explanation of his process:

This is what is sometimes called a "list" poem. It works well in this situation as I did not want to leave out any of the wonderful words and phrases that the participants wrote.

The first thing I did was to alphabetise the participants' phrases.

The next step was to decide where the smaller divisions (the stanzas) might go—some of this was determined by how many phrases began with a particular letter of the alphabet, and others just seemed naturally to belong together.

The final thing was the selection of the ten indented single words that finish each stanza, that reflect upon the feeling or motion of what is just above them on the page. These also make a lovely poem when read on their own, one that "sums up" the group's work.

Source Material:

Independence lost
Lost for words
Misunderstood and fragile
Anxiety oh dear oh dear
Connect with the inner spirit beyond the inabilities
Time slows down and remembering to truly be in the moment
I remember being mindful with my breathing as I consciously joined them in their reality
Often there was silence the room although their energy (a different kind of energy to when they were well) was very much still there

Unfinished business, unable to complete or explain
Stuck in a loop, repeating the same actions everyday
Unable to make them well again
Challenging symptoms and behaviour
A steep learning curve
Exhaustion leading to chronic fatigue
Frustration navigating the healthcare/social care system
Deep compassion
Remembering happier times
Needing to cope with boredom
Feeling of worthlessness
Frustration
Always on duty
Feeling restricted

6.1 ***What is your experience of having PCA or being a family member caring for someone with PCA?***

Still Me

Reinvent yourself—

A white cloth on the table

Give me a white plate

Looks like my food is floating

A sensory overload

Unclear. Unreal. Next?

A tunnel—where does it lead?

Bright sunshine? No. Fog

Hallucinations scary

Sometimes funny, confusing

Not mad at myself

Still me and always will be

Time to have a laugh

This tunnel—will roof collapse?

Will there be rats? Will it flood?

Wading through treacle

Dark and sticky—destiny?

Forget. Reinvent

Watching and listening—no

Time to have a laugh

A man enjoys clothes

Wears pyjamas over shirt

Escapes from the house

In tracksuit bottoms, odd socks

Flat cap, carrying slippers

Light to see forward?

Starting to rely on touch

Not sight—a struggle

Reducing my confidence

Still me and always will be

Foggy day—ah, well

Amazing music, recalled

TV, film—unreal

I try not to get angry

Sometimes more successfully

Forget. Reinvent

Frustration. Fear. Overload

Will there still be light?

PCA—unclear—what next?

Still me. I will always be

Poet's explanation of his process:

Senryu is an ancient Japanese form with the same origins as haiku, but focusing on observations of human experience rather than commenting on nature. When linked into longer poems, they become what is known as renga, adapted by non-Japanese writers using a pattern with groupings of three lines in five syllables, seven syllables, and five syllables again, then two lines of seven syllables each.

This writing group produced some really wonderful images—more images than narrative, some funny, some heart-breaking, all insightful—and the senryu/renga form emphasises both the shared experiences and the unique ones. I was not able to include every single word, but I was able to bring in every image.

Source Material:

PCA can be confusing
Hallucinations can be scary or sometimes funny
Put a white table cloth on the table and give me a white plate, it looks like my food is floating on its own above the table
Bright sunshine, oh no sensory overload, personal foggy day
Watching and listening a no go

Foggy day, ah well
Forgetting and constantly 'reinventing' yourself is a frustration
I try not to get angry with myself, sometimes more successfully than others!
I keep telling myself I'm still me and will always be
Responsibilities
Unclear
What next
Unreal
Not me
In a tunnel
Will it flood?
Will the roof collapse?
Will there be rats?
Where does it all lead?
Will there be light to see the way forward, or are we destined to forever wade through a sea of darker and stickier treacle
The man who showed me how to enjoy clothes wears a pyjama top over a shirt
a flat cap, odd socks
rolled up tracksuit bottoms
And escaped from the house wearing shoes, but carrying slippers.
Take every opportunity to have a laugh
Amazing recall with all my music
Reducing confidence
Struggle with tv and film
Starting to rely on touch instead of sight

7.1 ***What is your experience of having dementia or being a family member caring for someone with a dementia?*** [mixed rarer dementia group]

No One Would Believe Me

Do you have anxiety?
People are so rude --
no one would believe me: *heartbreaking,*
cruel disease, disaster, increasing difficulty

My most frustrating memory gap
is not being able to recall …
I am so, so depressed
there is no treatment to stop or slow up…

mad; furious; sad; lonely

I cannot find my wife,
she doesn't know she's lost.

Why is Mom having trouble walking?
Mom could hurt herself ...

I will not live to be an 'old' lady
I am not following the other lovely women in my family …

They say it's good to learn new things.
Anosognosia is not one of them.

the names of my former students and teacher colleagues
my most frustrating memory gap

Do you have anxiety?
tiring

Learn to forgive yourself when you become exasperated.

Mom was right: what do we do?
Learn to forgive yourself when you become exasperated.
We walk holding hands again, like when I was little.
How long will this last? I still see her.

Eyes closed now.
Her laugh is still hers.

We're all ok.
Learn to forgive yourself -
Her laugh is still hers.

Poet's explanation of her process:

The texts from the respondents had a lot of poetry in them. I printed the texts and cut them up into useful fragments. Then I tried various arrangements and started writing down the ones that worked immediately. Whole sections of the poem were already coming together. The themes of distress and worry were balanced by the theme of caring and the individualized, resounding voices of the respondents. The condition threatens erasure but the personal, evocative voices insist on love bearing witness.

I was able to use most of the respondents' words and I was very moved by all of them.

Source Material:

Disaster; Upset; Mad; Furious; Sad; Lonely; Tiring; Mentally, Emotionally, and Physically Exhausting
My most frustrating memory gap is not being able to recall the names of my former students and teacher colleagues.
I can not find my wife,
She doesn't know she's lost.
They say its good to learn new things.
Anosognosia is not one of them.
Learn to forgive yourself when you become exasperated.
I am so, so depressed that there is no treatment to stop or slow up the progress of Primary Progressive Aphasia
I am gradually experiencing the increasing difficulty in remembering the definition of some words, how to spell some words and how to properly pronounce some words
very sad that I will not live to be an 'old' lady and will die much earlier than I expected as I am not following the other lovely women in my family
if I can assist to be part of research for PPA to help find treatment/cures (maybe those +ve results won't happen while I am alive) but I would be pleased to contribute to help others with PPA in the future.
Heartbreaking
Cruel disease
No one would believe me
1. we're all ok.
2. do you have anxiety?
3. why is mom having trouble walking?
4. shit-mom could hurt herself...
5. fuck, people are so rude
6. double fuck. mom was right. what do we do?
7. how long will this last? i still see her.

We walk holding hands again, like when I was little.
Eyes closed now.
Her laugh is still hers.

Cohort 8.1 *What is your experience of having dementia or being a family member caring for someone with dementia?* [mixed rarer dementia group]

Changing

I am a member of a shitty club:
Life Changing

life changing

at the beginning I felt
somewhat lost and cheated
surprised and confused
frustrated
embarrassed
devastated

(life changing)

I needed to fight, be more patient
plans needed to change

I needed to find answers
sadness
lost memories

I needed to accept and become a better person
with hope anything was possible
honouring our vows
loving you in the present moment

doing the best I can
"in sickness and in health"

life changing

though I am a member of a shitty club
I needed to find answers, and
I felt at peace that I am doing the best I can

Poet's explanation of her process:

I am primarily a free verse poet, and the sound of words and poems is particularly important to me. In reading this group of responses, I was immediately struck by the prosody in what became line 1, which seemed to be trying to be a song, and therefore was looking for a refrain. "Life changing" is the underlying theme here, and became the refrain almost immediately. The responses shaped the poem into parts addressing the situation of change, looking into how it could possibly be handled, and trying to find courage while admitting that the situation is extremely difficult. The responses quite clearly were a song waiting to happen; my task was to find the song that was already there.

Source Material:
Devastated; Embarrassed; Frustrated; Surprised and confused
Sadness; Lost memories; Life changing
At the beginning I felt somewhat lost and cheated. Plans needed to change
With hope anything was possible.
I needed to accept and become a better person.
I needed to fight, be more patient and find answers - at first it was a hectic pace, as time passed I felt at peace that I am doing the best I can. I do however need to continue to work at being more patient. My spouse with FTD surprisingly is the best teacher.
member of a shitty club
honouring our vows of "in sickness and in health'
loving you in the present moment

Cohort 9.1 ***What is your experience of having FTD or being a family member caring for someone with FTD?***

Eleven

1.
life changing
horizon shrinking
energy zapping doors
closing
longing for this not to be

2.
who cares for me, myself?
where did all the friends go?

nobody drops by
they have no idea of the reality
the cruellest disease you have never heard of

3.
you never know what you are going to get
I go into my own dark space
overwhelming, exhausting
this fear for the future t
this trepidation for what's to come

4.
invisible
written off
scary, sad, helpless
I fight
I know I'll lose

5.
what's being lost
hard to accept
the world shrinking
slowly taking everything away
one bit at a time

6.
Strangers in the home
In your life
Frustrating, challenging
Deep love, unconditional
These strangers become your friends

7.
roller coaster of emotions
embarrassing to forget people's names
keeping focused on preserving my own identity
a heavy burden to carry
there is no one left

8.
harrowing, destructive, relentless
soul-sucking, sadistic disease
"look after yourself" they say

"you can live well with dementia"
robbed of being, cruelly, slowly

9.
protective, vulnerable
confusion, lack of understanding, sorrow
brain giving up
the people they once knew
witness apathy, loss of empathy

10.
"life is a box of chocolates"
crazy beast continual loving care, inspiring
appreciate each stage whilst you are in it
as the next brings more loss, more sadness

11.
the challenges, the pain
reactions from friends and family
forget events in the past
forget things you have committed to
slowly fading

The poet's explanation of his process:

As there were eleven participants in this session, and quite a lot of words produced in response to the prompt, a good deal of editing was necessary, lest the poem become overly long and unwieldly to read and absorb. I divided it into eleven stanzas of five line each, which enabled the inclusion of every person's ideas, cut and pasted for what I hope is maximum effect. This also gave the poem its title: *Eleven*.

This session included particularly powerful, expressive, and astonishingly honest reactions—a great deal of sorrow, fear, frustration, and fury, with only the smallest glimmers of acceptance or hope. Not easy to read, but a piece, I think, that could help readers with and without dementia to understand what this group of writers is experiencing at the moment. Thank you for the opportunity to work with your words.

Source Material:

it is an embarrassment to forget people's names, events in the past and things you have committed to

Riding the roller-coaster of emotions.
Keeping focused to preserve my own identity.
Continual loving care for both of us.
Horizon-shrinking,
Energy-zapping,
Inspiringly-Joyful....... bvFTD
I go into my own dark space
There is no one left
Who cares for me myself
UNCONDITIONALLY
Harrowing, despicable, destructive, relentless, soul-sucking, sadistic disease. beast.
FTD is a heavy burden to carry.
The cruellest disease you have never heard of'.
Life is like a box of chocolates .... you never know what you are going to get until bite into it !
Life changing
Crazy
Loss of empathy and ritualistic behaviour are the first signs of this disease
Checking out which pub the quiz is in tonight tomorrow the night after quiz quiz every night ......he gets to the pub at 6 pm for the quiz at 9pm ....he doesn't get to do the quiz he just gets drunk by 9 pm I pick him up and get him home to bed ...."how many pints have you had I say " ....I've had two f****g pints is always the answer
Reactions from his friends my friends and family......."look after yourself they say "...they never say I will take him to the pub and get him home for you to have a night off....they never say drop him off to my house and get your hair done "....they just say LOOK AFTER YOURSELF
Where did all the friends go ? No phone calls from work mates quiz mates, nobody drops by
It's hard to accept carers who are strangers into your life into your home .....then you get to realise these strangers are becoming your friends
Overwhelming
Exhausting
Scary
Sad
Helpless
Sorrow for what's being lost
Fear for the future
Trepidation for what's to come
Longing for this not to be
Doors closing, fading slowly. Becoming invisible and written off.
Brain has given up on me. I fight I know I'll lose.
Robs a loved one of their very being, slowly taking everything away, one bit at a time

People continue to say “ you can live well with dementia” yet they have no idea of the reality, the challenges, the pain of this cruel slow degenerative disease
As a carer, however frustrating and challenging it is, you have to appreciate each stage, whilst you are in it, as the next brings more loss and sadness
Feelings of protection, vulnerability and deep love for the person with dementia, as you witness their world shrinking – the confusion and lack of understanding of everything and the people they once knew.

10.1 ***What is your experience of having or being a family member caring for someone with a rare/young onset dementia?*** [mixed rarer dementia group]

Accept This Honour

Walking in a long dark tunnel --
Struggling to understand the journey –
Heart-wrenching to feel so helpless --
Hopeless against this unrelenting disease --
This train wreck happening before my eyes:

> She has the function of a toddler
> with the attitude of a teenager
> with all the guilt of being my mother…
>
> The joy of reading her her favourite poem
> and getting the same reaction
> like she hasn't heard it in
> forever…
>
> The sister I loved so dearly
> was being taken from me
> piece by piece ...
>
> Miss my partner
> even though he's physically still here,
> trying to be positive and know
> these moments with him won’t come again…
>
> I just hope she knows that even when I am frustrated
> I still love her so much ...
>
> If only there was a cure or something to stop this!
>
> The need someone who they can trust…

It’s YOU…ACCEPT this honour--
Show your patience, understanding,
unconditional love and dependability.

Accepting the inevitable
Tiny snippets of dappled sunlight

The poet’s explanation of her process:

Responses in this group worked very well in free verse, divided into descriptions of living with the condition and accounts of individuals. The general descriptions, collected together, suggested a way to offset the individual stories like the decoupled cars of a train, and then put everything back together again with the brave stoicism several respondents voiced. The responses were very poetical, touching, and successful expressions of the challenges of this condition, and provided the energy that drives this poem.

Source Material:

Show your patience, understanding, unconditional love and dependability.
They need someone who they can trust…it’s YOU…ACCEPT this honour
Struggling to understand the journey, the sister I loved so dearly, was being taken from me, piece by piece. Heart-wrenching to feel so helpless and hopeless against this unrelenting disease. If only there was a cure or something to stop this train wreck that was happening before my eyes.
- She has the function of a toddler with the attitude of a teenager with all the guilt of being my mother
- I just hope she knows that even when I am frustrated I still love her so much.
- the joy of reading her, her favourite poem and getting the same reaction like she hasn't heard it in forever.
isolating, especially during Covid
miss my partner (with YOD), even though he's physically still here
trying to be positive and know these moments with him won't come again
Walking in a long dark tunnel
Tiny snippets of dappled sunlight
Accepting the inevitable

11.1 *What is your experience of having PPA/FTD?* [this cohort consisted only of PLwRD and no carer-partners]

When Will I No Longer Be Me?

tiredness, frustration, disorganisation
thank God for the support group
    the most mundane things take the largest effort

don’t be ashamed of me
I realize my personality has changed
    what is the rate of my decline?

I can’t remember the shock but there must have been
run away and hide
    when will I no longer be me?

I have some words in a day
and then they don’t come through the next day
    the most mundane things take the largest effort

letter, number and word-finding difficulty
then curiosity and call to action take over
    what is the rate of my decline?

perpetual grief, rapid fluctuations in mood
I don’t understand this
    when will I no longer be me?

Who can help me?
I often know the words in my brain
    the most mundane things take the largest effort

I am energised and happy
participating in mealtime conversations with friends, I mostly listen
    what is the rate of my decline?

when I have a strong opinion, I try to explain my point of view
learning about myself and a brighter side of this world ever since
    when will I no longer be me?

empowerment to create understanding around dementia
let's build that old-fashioned village again
    the most mundane things take the largest effort

my first thought was, "Thank God it's me"
anger, emotional upset, lack of control
    what is the rate of my decline?

heard of dementia, heard of Alzheimer's
never heard of FTD/PPA
    when will I no longer be me?

challenging—I spell a word: p-l-e-a-s-e
then a few minutes later "please" come out
    the most mundane things take the largest effort

new friendships, love and support, acceptance and patience
surrounding love—these people have lifted me
    what is the rate of my decline?

am I going to die soon?
the world needs to know and see
    when will I no longer be me?

The poet's explanation of his process:

This particular prompt elicited a wide variety of heartfelt fears, questions, and fragments of personal stories, all of which have found a place in the final piece. In putting together a longer poem, such as what was needed in this instance, structure becomes particularly important. I carefully reviewed past RDS poems as well as poetry anthologies on my bookshelves before deciding on an adaptation of an old form, the ***tercet***, one used by poets as diverse as Shelley in the early 19th century and William Carlos Williams in the mid-20th.

The name comes from the Latin for "third" and consists of stanzas made of three lines each. Often there is a rhyming pattern, but in this case the emphasis is on repetition—there were three lines in the source material that seemed to sum up the mood and served to group the responses into potent statements of personal

experience, with each repetition yielding a slightly different effect. The overall form features the three lines of a tercet, but the repeating lines create an additional tercet-based framework, a "super-tercet," on which to build the poem.

Source Material:

I cant remember the shock but there must have been.
My first thought was "thank god it's me.
My second is, dont be ashamed of me, the world needs to know and see.
Then curiosity and call to action took over.
I have been learning about myself and a brighter side of this world ever since.
I am energised and happy.
Let's build that old fashion village again.
perpetual grief
surrounding love
run away and hide
I realized my personality has changed.
When participating in lunch/dinner conversation with friends I mostly listen.
When I have a strong opinion about a topic I try to explain my point of view.
I don't understand this, never heard of FTD/PPA. Head of Dementia , heard of Alzheimer
Am I going to die soon?
Who can help me?
Thank God for the support group, these people have lifted me
the most mundane things take the largest effort
what is the rate of my decline
when will I no longer be me?
Empowerment to create understanding around dementia
Tiredness
Frustration
Acceptance and patience
Emotional upset
Anger
Lack of control
Rapid fluctuation in mood
Disorganisation
Challenging
Letter, Number and word finding difficulty
New friendships
Love and support
I have some words in a day and then it doesn't come through the next day. I often know the words in my brain and will say it as
p-l-e-a-s-e, then a few minutes and please comes out in my brain.

12.1 *What is your experience of having a rare or young onset dementia or being a family member caring for someone with a rare or young onset dementia?* [mixed rarer dementia group]

Out Here in the Rain

"We are not the walking dead"

I'm stuck here with a rare disea … eeeze

devastating

terrifying

heart breaking

frustrating

The physicians don't have a clue, who?

very maddening

very stressful

very sad

I'd be stuck in last century

sitting here clueless

fear

stigma

You blessed me with a nursing degree

opportunity

education

support

understanding

Becoming and blossoming into something different

yet beautiful

Finding a deep sense of self

freeing

comical

Hours fly by like minutes

faces I ought to remember

no longer provide me names

I read and read about how to ease the pain

but none of the doctors can agree … ee

leaving me out here in the rain

with all the pain

Explanation of the poet's process:

This free verse poem responds to the musical suggestion of one respondent and becomes a song, with opening lines followed by a chorus. I imagine this as the "star" singing the openers and the back-up singers doing the italics. Although the subject matter is very serious, the undercurrent of light-heartedness comes through in the arrangement. Readers get to make their own music.

Source Material:

Devastating
Terrifying
A great sadness
Becoming and blossoming into something different yet Beautiful
Finding a deep sense of self
Freeing
Heart Breaking
Frustrating
Comical
Hours fly by like minutes
And faces I ought to remember
No longer provide me names

Caring for a family member with young on set dementia is:
Very sad
Very maddening
Very stressful
Fear, understanding, education, stigma, support, opportunity and my phrase is "We are not the walking dead"
Ooooouu baby, baby,
I'm stuck here with a rare disea. eeeze
and the physicians don't have a clue,
who?
Oouuu baby, baby,
You blessed me with a nursing degree …ee
Or I'd be stuck in last century y
With sitting here clueless with my MD
Ou baby, baby,
I read and read about how to ease the pain
But none of the doctors can agree ee
Leaving me out here in the rain
With all the pain

13.1 ***What is your experience of having a rare or young onset dementia or being a family member caring for someone with a rare or young onset dementia?*** [mixed rarer dementia group]

How May I Help You?

Use short sentences:

LIFE IS TOO

BLEEDING

TEARS LIKE RAIN

I don't want

PAIN

risk anger FURY

I am angry.

I am trying to cope

as normal as possible

and still be able to communicate with people.

Thoughts over time after learning of the diagnosis:

confusion – frustration – patience – disbelief – skepticism – compulsive behaviours -- unknown uncharted territory -- cascading loss – isolating – endless –wrenching.

I never thought a disease could be so horrible

and have such a dramatic impact on a family:

loss, loss of myself, my essence, my work, my driver's license, my job, my body,

friends, love of husband because he feels overwhelmed by extra housework.

obey

recoiled in fear

would not

could not

cruel indifference

he lied

MENTAL HEALTH

I am sad.

Sadness is my daily companion.

People don't understand a rare form of dementia.

Even professionals seem at a loss

and are slow to respond to a 'rare' form of dementia –

they seem at a loss of what to do.

I don’t want

she screamed

guilty

unclothed

FIRE

remorse

When will this end?

Our dreams are shattered

and we will never be the same again.

Scared.

Fear of the future.

Fear and uncertainty about what’s coming next.

I will never get the chance to say good bye.

Sorrow for my son and granddaughter when I'll be gone.

I don’t want

love

tears like rain

life is too

I try to remain in the Present,

grateful that I have notice of my condition.

Love keeps me holding on,

empathy.

We live in the second.

Everybody has been pretty good to me.

I am strong & supported.

Hope holds a spark

that beckons me.

TEARS LIKE RAIN

The poet's explanation of her process:

Responses in this group expressed the struggles of living with early onset dementia in very powerful language. The idea and instruction "use short sentences" suggested how to contrast brief comments, wrung out of pain and concern, with longer statements about the condition. The poem has bursts of words that in their incompleteness describe the difficulty of conveying meaning, and the deep grief of the suffering. The poem proves that language does have the power, albeit imperfectly, to try to describe the condition.

Source Material:

MENTAL HEALTH
LIFE IS TOO
She screamed
I don't want
I don't want
I don't want
Pain
Guilty
Unclothed
Anger
Recoiled in fear
FURY
Risk
Remorse
Obey
He lied

Would not
Could not
Love
Cruel indifference
FIRE
BLEEDING
TEARS LIKE RAIN
I am sad.
I am strong & supported.
I try to remain in the Present.
Everybody has been pretty good to me.
I am angry
I am trying to cope as normal as possible and still be able to communicate with people.
Confusion – frustration – patience – disbelief – skepticism – compulsive behaviours - empathy – unknown – scared - long history of abusive behaviour – Have the symptoms been present for over 20 years, and have expressed themselves as symptoms of various mental illness and personality disorders?
Uncharted territory
Cascading loss
Fear and uncertainty about what's coming next
Sadness is my daily companion.
Hope holds a spark that beckons me.
Love keeps me holding on.Isolating
Endless
Wrenching
* how may I help you?
* use short sentences
* when will this end?
- People don't understand a rare form of dementia.
- Even professionals seem at a loss and are slow to respond to a 'rare' form of dementia
- they seem at a loss of what to do.
Thoughts over time after learning of the diagnosis:
"disbelief, confusion, fear, anxiety, frustration"
1) We live in the second.
2) I will never get the chance to say good bye.
3) Our dreams are shattered and we will never be the same again.
4) I never thought a disease could be so horrible and have such a dramatic impact on a family.
- Loss
- Loss of myself, my essence, my work, my driver's license, my job, my body, friends, love of husband because he feels overwhelmed by extra housework.

- Fear of the future
- Sorrow for my son and granddaughter when I'll be gone
-Grateful that I have notice of my condition compared to someone who dies suddenly

---

5.2 *How would you describe PCA to a friend or family member?*

From the poet: This type of poem is known as a "foursquare," and its flexibility allows it to be read vertically, in columns, or horizontally, line by line, or even diagonally if you like.

Bewilderingly So

PCA is cruel	it takes your mind
steals your futures	steals your dreams
heart-breaking to witness	a potpourri of symptoms
a badly shaken cocktail	especially cruel
reality can't be right	you're living it
frustrating, and you know it	angry, helpless
the paving stone like a hole	diminishing visual awareness
no handles, no keyboard	the fog of dementia
rollercoaster of emotions	independence and language lost
the impact as it progresses	no way to retrieve it
steals the person you love	everyday practical skills
bewilderingly so	a few precious moments shine through

The poet's explanation of his process

When I saw that there were four participants who responded to this particular writing prompt, I started experimenting with the foursquare model of crafting a poem. It is inspired by the traditional schoolyard game of four players standing in the corners of a grid and taking turns to bounce a ball quickly amongst themselves. In this case, though, there's no rubber ball crisscrossing and ricocheting, but intense

ideas, honest emotions, and profound and insightful descriptions, arranged into groups of four and those groupings arranged into a larger foursquare shape.

Given the storytelling nature of some of the responses, it wasn't possible to include every single word of the source material, so I worked with the selection of phrases and fragments that fit together well in at least two different adjacent positions. The images harmonise or contrast differently depending on the direction one reads, altered by new juxtapositions.

Source Material:
PCA is cruel – it takes your mind … and you know it.
You know your reality can't be right but you're living it – setting down a cup on a table that's actually two feet to the left so it falls, the paving stone that looks like a hole, the wallpaper that appears to be on the front of your husband.
And as PCA progresses, the brain becomes like a filing cabinet with no drawer handles. Or a hard drive with no keyboard. You know the data is there but you've no way to retrieve it.
Frustrating
Angry
Helpless
Steals your futures
Steals your dreams
Steals the person you love
Heartbreaking to witness the impact as it progresses
It seems especially cruel, by diminishing visual awareness and ability to speak as well as everyday practical skills, like writing
Independence and interests, including language, become lost…bewilderingly so if without a formal diagnosis... and the right support
A rare neurological condition, similar to that experienced by Terry Pratchett
A badly shaken cocktail of neurotransmitters = a potpourri of symptoms & a rollercoaster of emotions
A few precious moments when, even through the fog of dementia, her sense of humour shines through…

6.2 ***How would you describe PCA to a friend or family member?***

Where to Begin

Where to begin?
    Brain blindness
    Vision is darker. That's not a rubber mat?
    I can see it, but at the same time I can't see it.

Confusing, disorientating.
Good days and foggy days
Frightening
Where to begin?

Which way round does this go?
Things aren't where they appear to be
I wish you could see through my eyes
Invisible dementia
Misdiagnosed and misunderstood
Getting confused more often
How to describe something
No more words
Which way round does this go?

Why can’t I find things?
I wish you could see what I see
It’s a black hole
I can’t do anything I used to be able to do
Eyes are okay in themselves
No more driving
Staying positive as possible
How to begin?
Why can’t I find things?

Where is the top or the bottom?
When I struggle to understand
Word searching
Things aren't where they appear to be
The brain effects the eyes
Embuggerance
No more words
Misunderstood
Where is the top or the bottom?

Where is the top or the bottom?

Why can't I find things?
Which way round does this go?
Where to begin?

The poet's explanation of his process

Reading the group's responses, I was immediately struck by the questions—the feeling they gave of being uncertain, of wishing for answers and clarity that are never entirely provided—and I noticed that, although similar, the questions never repeated exactly word-for-word.

This became the starting point. Repetition is a key component to poetry, along with rhythm and word-play—tools used to heighten the reader's awareness of the language being used. While there are some poetic forms that rely quite heavily on repetition, like pantoums and villanelles, none of them really suited this collection of responses, so I worked at crafting a structure that would. It also felt important to make sure to include as many of the original words as possible—fragmented, juxtaposed so that no one writer's words dominate or end up next to the places where they began.

I think that this a poem that benefits very much by being read aloud. Every time a repeated line is heard, its impact changes.

Source Material:

The brain affects the eyes
Eyes are ok in themselves
Misdiagnosed and misunderstood condition
Brain blindness
"Embuggerance" (Terry Pratchett)
Invisible dementia
Frightening
It means I can't do anything I used to be able to do
Vision is darker
No more words (reading, writing, understanding)
Word searching!!!!!!
No more driving!!
I can see it, but at the same time I can't see it.
I wish you could see through my eyes!!! and see I what I see.
That's not a rubber mat? "IT'S A BLACK HOLE".
Confusing, disorientating Why can't I find things?
Things aren't where they appear be.

Which way round does this go? Where is the top or the bottom?
PCA to me, is having, good days and 'foggy' days, getting confused more often, whilst staying as positive as possible
Where to begin?
How to begin?
How to describe something, when I struggle to understand

7.2 ***How would you describe your or your family member's dementia diagnosis to a friend or family member?*** [mixed rarer dementia group]

Unwavering braveness
Brain blindness.
Extreme turmoil.
An excruciatingly slow
drip
of profound
loss.

Unwavering
braveness.

A time
when old memories
get lost
but can be found.

You easily see that she is
not the same
person you remember
but she believes
that she has not changed
that she is still
the same person
she has always been.
She may not
understand the nuance
of the situation

and say or do
something
out of place.
When you speak
to her she may not know
a word
you use.
She will ask
you
the meaning
of that word.
You often have to
repeat
a question and
her answers will
likely be slower
than before.

PPA can
progress
slowly.
As it is progressing with
me I am finding:
I have to think more about
how to pronounce words
correctly, to spell
correctly, to remember
the definition of a word.
My short term
memory is not as good
either -- so I record
a lot of things
to make sure I
remember; for example,
what to do, what has happened

I can get very sad
some days
about the progress, but then I try
to remember
how blessed my life
has been – I know
I will not be living as long

as many of my relatives/parents –
but try to focus
on how wonderful
many parts of my life have been

PPA is very depressing, to know
that, at this point,
there is no cure/treatment
for it. I am MOST interested
to be involved in research
if I can
to help anyone who gets PPA.

The poet's explanation of her process:

Responses in this group were about the difficulties of trying to communicate about the condition, of trying to find and remember words, and make meaning for others. Therefore, I broke up the statements into smaller parts and floated them in the middle of the page to express the fragility of the attempts to communicate. I hope the result is a beautiful and delicate form, with words carrying extra emphasis from their temporary isolation from each other.

Source Material:

Disaster; Upset; Mad; Furious; Sad; Lonely; Tiring; Mentally, Emotionally, and Physically Exhausting
My most frustrating memory gap is not being able to recall the names of my former students and teacher colleagues.
I can not find my wife,
She doesn't know she's lost.
They say its good to learn new things.
Anosognosia is not one of them.
Learn to forgive yourself when you become exasperated.
I am so, so depressed that there is no treatment to stop or slow up the progress of Primary Progressive Aphasia
I am gradually experiencing the increasing difficulty in remembering the definition of some words, how to spell some words and how to properly pronounce some words
very sad that I will not live to be an 'old' lady and will die much earlier than I expected as I am not following the other lovely women in my family
if I can assist to be part of research for PPA to help find treatment/cures (maybe those +ve results won't happen while I am alive) but I would be pleased to contribute to help others with PPA in the future.

Heartbreaking
Cruel disease
No one would believe me
1. we're all ok.
2. do you have anxiety?
3. why is mom having trouble walking?
4. shit-mom could hurt herself...
5. fuck, people are so rude
6. double fuck. mom was right. what do we do?
7. how long will this last? i still see her.
We walk holding hands again, like when I was little.
Eyes closed now.
Her laugh is still hers.

8.2 ***How would you describe the diagnosis to a friend or family member?*** [mixed rarer dementia group]

There Is So Much I Could Say

FTD is a journey,
a terminal degenerative brain condition,
a slow and painful death I encounter each day,
a condition which steals an individual's personality.

There is so much I could say:
Frustrating when you can't remember
words
or things --
her friends no longer call as before.

There is so much I could say:
common household tasks are difficult,
worried if husband
not available to take care of
business,
taxes,
driving car,
house work.

There is so much I could say:
PPA as changed our retirement plans,
worried about being a bother
or a problem at times
or in the future.
The chapters of our retirement will be
different than the plans we had made.

There is so much I could say:
it demands continuous learning.
It requires extra sprinkles of patience
for your loved one who has limitations
with communication and planning.
My husband is my best friend and soulmate.
I am a lucky woman to have him in my life.
He treated me like a queen for over 30 years
and he is still my knight in shining armour.

There is so much I could say:
Live each day as that is the only guarantee you have,
that is how I live life now!
There is so much I could say!

The poet's explanation of her process:

Respondents in this group reported on the difficulties and frustrations of trying to express their experience of the disease, yet they were conveying important information and illuminating their feelings. I grouped similar ideas together and worked with the theme that they were successfully doing what they felt they Couldn't do – speak to the limitations of the condition. I broke up the lines to indicate distress, and repeated "There is so much I *could* say" as meaning "There is so much I *am* saying", with more to be said in future. The effort to communicate is working, and works in poetical space.

Source Material:

Frustrating when you can't remember words or things
Worried about being a bother or a problem at times or in the future Worried if husband not available to take care of business, taxes, driving car, house work

PPA has changed our retirement plans
her friends no longer call as before
common household tasks are difficult
FTD is a journey. It demands continuous learning
It requires extra sprinkles of patience for your loved one who has limitations with communication and planning
There is so much I could say
My husband is my best friend and soulmate. I am a lucky woman to have him in my life. He treated me like a queen for over 30 years and he is still my knight in shining armour, even though the chapters of our retirement will be different than the plans we had made
Live each day as that is the only guarantee you have, that is how I live life now!
a condition which steals an individual's personality
a terminal degenerative brain condition
a slow and painful death I encounter each day

9.2 ***How would you describe FTD to a friend or family member?***

Description

From the poet: "*Description*" is in a form that is sometimes called a "stack" poem, as the words are arranged as just one or two words in columns—these can be read horizontally or vertically, and the meaning and imagery changes depending on the direction. If you think of the words as coins in a stack, each one is valuable in itself and when placed with others becomes important to the total sum.

not Alzheimer's rarer destroyer crazy sadness
different altering exasperating changed
apathetic empty distressed rituals
hypercritical atrophy by default blackouts
frontal lobe diagnosis loss poor judgement
destructive illness consuming classic
symptoms

no insight aggressive aggravating memory loss
no control behaviours mutate erode
eats away everything heart-breaking soul-destroying
love confusion hard work disappears
different apathy anger alone

frightening repetitive unwell for help

you thought empathy in depth awful
you knew concern together confirmed
comfort language personality never heard
once-loving memory hides behind own world
confusing one knew thoughts perceived norms
disinhibited suffering devastating lacking

The poet's explanation of his process:

The prompt, *How would you describe FTD to a friend or family member?,* elicited some extraordinarily eloquent and emotional responses, some exceedingly painful and all of them heartfelt and significant to the understanding of researchers, carers, and the general reader for the understanding of this disease.

Approaching the responses as a poet, the sheer number of words, as well as the story-telling nature of some of them, presented a challenge of condensing and arranging. In this case, I was not able to include every word that every respondent wrote, but I think that in selecting key words from each person and arranging them in this form that a unifying poem has coalesced.

Some stack poems feature columns that are fifteen or twenty words tall, but in this case I kept each stack to six, partially to intensify the effect of the responses and partially because the form echoes the oracular hexagrams of the I Ching, the ancient Chinese *Book of Changes*.
A column of six single words equates to the hexagram for "force," for instance, while one that follows the pattern of 1-1-2-2-2-1 translates as "persevering." I'm not making claims that this poem encapsulates the wisdom of the ancients, but it is interesting to consider the philosophical possibilities of fortuitous arrangements, and how the stacked words might contradict or reinforce the traditional meanings.

Here is a Wikipedia link if you would like more information about the I Ching and the sixty-four hexagrams: https://en.wikipedia.org/wiki/Hexagram_(I_Ching)

Source material:

It's not Alzheimer's
The cruellest dementia you have never heard of.
A rarer dementia associated with personality, behaviour and language.

Repetitive behaviours and loss of empathy.
“The Destroyer of Lives”
Crazy sadness loss
Frighteningly different to what you thought you knew.
Life altering
Exasperating!
The first thing I noticed that changed in my husband was apathy, he was always a hard working man got things organised without any effort, he became towards getting ordinary chores done and would look through me empty of concern for me when I asked for help.
I was unwell and required surgery, again he looked through me when I was in pain or distressed I felt so alone in my hour of need . We had been together for over many years and always comforted each other. After I had had the surgery he was more concerned about the fact that I had moved the mode m from my bedside table shining in my face than how I was.
He had rituals getting the newspaper sitting and reading every page. Planning a night out to a quiz for as many nights of the week as he could. Going to the quiz at 6pm and the quiz started at 9pm consequently drinking for 4 hours consuming many more pints of beer per night.
He became hyper critical about different religions race and other peoples enjoyment such as football which he called mindless tribalism.
He began drawing out money from a joint account which was used for everyday bills one month drawing out £600 pound just for drink.
When he was diagnosed which was by default, after a brain scan he had when he was suffering from blackouts showed frontal lobe atrophy. I paid to see a neurologist who confirmed the diagnosis.
When I got home and look more in depth at the illness it was as if he walked out of the computer screen towards me, the loss of empathy, apathy, poor judgement, loss of organisation skills we listed as classic symptoms of this awful destructive illness. It was a relief to me that my once loving caring husband and father of my children had no insight and no control over his thoughts or behaviour.
And then the loss of memory starts to happen and your once strong loving friend and husband becomes your child.
A neurological disease that does not follow the perceived norms of Dementia of memory loss and only for the older person but far more aggressive and devastating as it affects behaviours and personalities of younger people it ways that make them disinhibited lacking empathy and insight and carry out very anti social and often unkind actions.
A disease that hides behind personality traits and and explainable behaviours at first till it begins to mutate and erodes everything you thought about that person and your relationship with them
A disease that eats away the soul of the person and everything they are or who they had wanted to be.

Devasting
Soul destroying
Heartbreaking
Confusing
Love confusion and anger as the mother one knew
disappears into her own world

10.2 ***How would you describe your family member's diagnosis to a friend or family member?*** [mixed rarer dementia group]

I Know You Have Noticed

My husband is a little different this year --
we received a diagnosis of dementia ...
it's heartbreaking --
the thought of this
heart-wrenching journey
breaks my heart in two.
Disbelief washes over me!
Web of words fading.

Her mind is cloudy.
She is slowly slipping away;
one function disappears at a time.
She is trying to remember her day
and the people around her,
like when you try to remember
a dream the details are all fuzzy.
Emotions might be deep inside
where you can't see them.

Safe when in his peaceful world
emotions might be deep inside …
Savour every minute shared together
as it is the memories made
that will help
through the darkest times.

The poet's explanation of her process:

This poem balances suggestions of two individual situations in which the difficult outside contains "emotions … deep inside". The poem calls on onlookers to pay deep attention in their noticing, and to retain memories made by noticing for the future. By noticing, onlookers can see the universal in the individual, and retain some comfort that will be needed.

Source Material:

(To very close friends) I know you have noticed my husband is a little different this year; we received a diagnosis of dementia….
Disbelief washes over me! The thought of this heart-wrenching journey breaks my heart in two. Savour every minute shared together as it is the memories made that will help me through the darkest times.
She is slowly slipping away, one function disappears at a time. It's heartbreaking.
Her mind is cloudy. She is trying to remember her day and the people around her, kinda like when you try to remember a dream the details are all fuzzy.
Web of words fading.
Safe when in his peaceful world.
Emotions might be deep inside where you can't see them.

11.2 ***How would you describe PPA/FTD to a friend or family member?*** [this cohort group only consisted of PLwRD and no carer-partners]

Tumbling, Jumbling

First of all, I have PPA
An indescribable experience
My mind plays tricks on me
I forgot the name of a shop
People's names and places we travelled to
I can understand the conversation
But I have difficulty in speaking

T U M J U M
B LE B L E

An unexpected challenge
I thought it was just me being so busy at work
It wasn't just one shop
Increasing opacity between me and the world
I get so tired just doing ordinary things
Please give me time to respond
To your comments and your questions

T M L J M L
U B E U B E

it happened again—I forgot the names of animals
I asked for an MRI brain scan
I got the diagnosis after assessments
Don't review the symptoms on Google
It's so scary to see it and goodness very depressing
My thoughts, words and actions have been put into a tumble drier
On spin mode to jumble and mix everything up

U B E J B M
T M L L U E

Now? Things are the same—it's just about animals and places
I have wonderful support—groups, one-to-ones, a family
I can drive, cook, garden, shop, walk the dogs, gym classes
I can remember what I need to do
But—Tumble. Jumble
Learn to accept
I am drifting away from you

The poet's explanation of his process:

When I first read this set of responses, two words leapt out at me and seemed to set the tone for the poem—indeed, almost giving me clear instructions that this was going to be a piece about rearranging and juxtaposing different participants' images and emotions—the "jumble." Additionally, I wanted to re-create the feeling of not being entirely in control—the "tumble."

This poem incorporates aspects of "concrete" poetry—not that it's made of cement, but a method of using the shape, size, and positioning of certain letters in order to reinforce the overall mood. In this case, I was attempting to reflect the lived experiences of the participants, and so the large-type words become more difficult to decipher each time the reader encounters them—we know what they say, but it's harder to figure them out as the poem progresses.

Source Material:

PPA. ... An unexpected challenge
An indescribable experience
Learn to accept
Sometimes it is as if my thoughts, words and actions have been put into a tumble drier, put on spin mode to jumble and mix everything up
I'm drifting away from you
My mind plays tricks on me
I get so tired just doing ordinary things
increasing opacity between me and the world
I forgot the name of a shop and thought it was just me being so busy at work !
It happened again and it wasn't just one shop , it was people's names and places where we travelled to.
I then forgot the names of animals. So, I asked for an MRI brain scan. Thats when I got the diagnosis after assessments as well.
Now? things are the same- it's just about animals and places. I can drive , cook, garden, shop, walk the dogs and do lots of Gym classes and remember what I need to do.
Don't review the symptoms on Google as it's so scary to see it and goodness very depressing
I have wonderful support, groups , 121's and a family.
I would say that, first of all, I have PPA. I can understand the conversation, but I have difficulty in speaking.
Please give me time to respond to your comments and questions.

12.2 ***How would you describe your type of dementia to a friend or family member?***
[mixed rarer dementia group]

Why I Repeat the Same Question at Random

You’ve been told there's something out of tune, in my muddled brain,
slow brain death, with physical appearance often not changing,
a rollercoaster ride,
confusion that overwhelms,
insidious,
heart breaking to see a vivacious intelligent person decline in this way.

Why I repeat the same question at random:
how can one word, de-ment-ia,
erase random notes on a page of a life lived in harmony, together?
Why does one, simple word change the dynamics of a life lived?

Why I repeat the same question at random:
dementia is a disease that tears at your heart;
to watch someone you love beyond words
become someone you don’t know
is terrifying, sad and maddening;
you can't cope with why I can't cook, or drive;
the loneliest place I have ever been,
the longest goodbye,
the “I’m not gonna miss you “effect.
And you just don't see me anymore.

Can you see me? I am here!

Dementia = Clarity.

It's ok here,

scary but joyful,

shocking but acceptable --

I have made friends with my dementia,

can live well but must do the work:

compassion

understanding

respect

peacefulness

inner strength.

Can you see me? I am here!

still here

still alive

still able to contribute

Why does one, simple word change the dynamics

of a life lived?

The poet's explanation of her process:

The responses in this group included the idea of a random but profound question that is repeated, which became the organizing principle of the poem: asking questions that can only partially be answered by personal accounts, definitions,

further questions. The poem offers two versions of living with dementia – the puzzling, sad side, and the attempt to find some positive aspect. The poem emphasizes the essential wish of respondents to be seen and heard as they try to explain living with the condition.

Source Material:
Shocking but acceptable. Scary but joyful. Can live well but must do the work.
"You've been told there's something
Out of tune, in my muddled brain
But you can't cope with why I can't cook,
Or drive; why I repeat the same question
At random; confusion that overwhelms
And you just don't see me anymore
Why does one, simple word
Change the dynamics of a life lived?
How can one word, de-ment-ia,
Erase random notes on a page
Of a life lived in harmony, together?
Can you see me? I am here!"
Slow brain death, with physical appearance often not changing, the longest goodbye, "I'm not gonna miss you "effect.
The loneliest place I have ever been
Dementia = Clarity, Peacefulness, Inner strength
It's Ok here, I have made friends with my dementia
Thank you for the opportunity to participate
Respect understanding still able to contribute still alive still here compassion
Dementia is a disease that tears at your heart , to watch someone you love beyond words become someone you don't know is terrifying, sad and maddening
Insidious
Heart breaking to see a vivacious intelligent person decline in this way.
A rollercoaster ride

13.2 ***How would you describe your type of dementia to a friend or family member?*** [mixed rarer dementia group]

Who Our Loved Ones Used To Be

Dementia doesn't care how old you are.

No cause; no cure.

Annoying to others, devastating for us.

A death sentence, no hope, dreams shattered, live in a maze till you die.

A disease that steals the essence of who our loved ones used to be.

Our life plans are over.

It is what I thought was going on.

I am not sure if I can do this.

Dementia is a term for symptoms of a diseased and dying brain.
Degenerative.
Starts with atrophy in the visual processing and spatial part of the brain.
Symptoms differ for each patient
depending on which area of the brain is affected.
Things are not the same, but don't be afraid to talk about it.
Know the signs to watch for, but remember
they can be different and will change over time.
Dementia is a disease like any other and there are many kinds.
Learn the difference.

Memory loss is not the main loss:
choking often, falling, loss of balance;
not able to make any decisions big or small on most basic tasks;
loss of not being able to accomplish what used to be second nature;
loss of being able to organize or focus on anything;

slowness in all we do.

Eroding our spirit, our personality, our own self.

She is still the same person, but is not able

to do the things she used to do because of the disease.

That's why we encounter people who don't believe it's dementia –

it's the everyday necessary things that are not obvious to you.

I would tell them I have early onset Alzheimer's and I am not in any pain.

But still I remain grateful for what is left

(so grateful to experience the greatest joy of having a granddaughter).

Be like the Magi - the best three gifts you can give

are patience, kindness and time - they are true gifts of love.

Be willing to support.

It's okay to do the things to keep me safe

even when I don't ask.

The poet's explanation of her process:
This poem describes the essence of how dementia affects bodies and lives, and alludes to the changes in the essence of those who suffer with it. The tone is dry and impersonal as difficulties are enumerated, but becomes more tender with references to real people and their real-life experiences.

Source Material:

Degenerative
Starts with atrophy in the visual processing and spatial part of the brain
No cause; No cure
Dementia is a term for symptoms of a diseased and dying brain.

Dementia doesn't care how old you are.
Symptoms differ for each patient depending on which area of the brain is affected.
Our life plans are over.
It is what I thought was going on.
I am not sure if I can do this.
I would tell them I have early onset Alzheimer's and I am not in any pain"
A death sentence, ho hope, dreams shattered, live in maze till you die
One day you are going to hug your last hug, kiss your last kiss and hear someone's voice for the last time, but you never know when the last time will be, so live every day as if it were the last time you will be with the person you love. Author unknown.
Know the signs to watch for, but remember they can be different and will change over time.
Things are not the same, but don't be afraid to talk about it.
Be willing to support and it's okay to do the things to keep me safe even when I don't ask.
Dementia is a disease like any other and there are many kinds. Learn the difference.
She is still the same person, but is not able to do the things she used to do because of the disease.
Be like the 'Magi' - the best three gifts you can give are patience, kindness and time - they are true gifts of love.
Memory loss is not the main loss
That's why we encounter people who don't believe it's dementia, it's the everyday necessary things that are not obvious to you
Not able to make any decisions big or small on most basic tasks. Choking often, Falling, loss of balance
Loss of not being able to accomplish what used to be second nature
Loss of being able to organize or focus on anything
Slowness in all we do
Annoying to others, devastating for us
Eroding our spirit, our personality, our own self
But still I remain grateful for what is left
So grateful to experience the greatest joy of having a granddaughter
I would describe dementia to them as a disease that steals the essence of who our loved ones used to be.

5.3 ***What does support mean to you?*** [PCA cohort group]

Truth: A Fib

myself
emotional

not dismissed
both
ready

care

myself
for somebody
concerns and observations
on call
understanding
compassion

expertise

advice

being there
understanding and compassion
at practical and emotional levels

ready to help someone who's about to fall

we're not alone in this
togetherness beyond family
constructive responses

knowledge
help

unexpected
relevant
affirm decision
talking problems through
loved one
myself
longer

specific
practical
being there
not

alone

The poet's explanation of his process:

This poem is a fib—which has nothing to do with telling the truth, and everything to do with an Italian mathematician of the 11th century.

Leonardo of Pisa, also known as Fibonacci, was the first western European to explain the series of numbers that we now know as the Fibonacci sequence, although they had been identified earlier in Indian mathematics and used as early as 200 BC in Sanskrit poetry. In the sequence, each number is the sum of the two preceding ones—0, 1, 1, 2, 3, 5, 6, 13, 21, 34, 55, 89, etc—and it can be used to describe features of the natural world like the double spiral pattern of seeds growing in sunflower heads, the way that pinecones twist, and the shape of the chambered nautilus.

When using the sequence to create poetry, one can choose to count either syllables or words. I found that counting by words was more useful this time. For this poem, I first sorted the original responses into groups, so that I had a number of single words, a few less doubles and triples, only a few fives and only one that was eight words long. The poem wasn't quite working as a single long stanza, so I broke it into smaller, mirror-image fibs, each one following the pattern of small to large and back to small. Several of the single words repeat more than once—they just cried out to be heard again.

Lines – sorted by number of words: In developing the poem, I first took participant's words and sorted them into groups, seen immediately below

1.
help
care
advice
myself
expertise
knowledge

2.
the unexpected
no longer
on call
constructive responses
being there
for somebody
affirm decision

3.
talking problems through
my loved one
understanding and compassion
specific and relevant
concerns and observations
not being dismissed
togetherness beyond family

5.
a practical and emotional level
we're not alone in this

8.
ready to help someone who's about to fall

My impression of the original responses is that the participants have used the prompt to describe a variety of positive, satisfying experiences. It's a glimpse for the reader into a very personal set of truths—so that the pun of the title stands out.

Source Material:

Togetherness beyond family
Understanding
Compassion
We are not alone in this
Being there for somebody
Help to affirm decisions
Ready to help someone who is about to fall
Specific and relevant knowledge, expertise, understanding and compassion
constructive responses
concerns and observations not being dismissed
Help, care and advice, on both a practical and emotional level, for my loved one and… myself!
No longer on call
Help with the unexpected
Talking problems through

6.3 ***What does support mean to you?*** [PCA cohort group]

SUPPORT

enablement, a listening ear

the spine, the confidence

the lighthouse in a storm

someone to catch you when the tsunami hits

SUPPORT SUPPORT

caring, kicking ass, holding my husband up

when I just want to talk

I'm not alone

I could not manage

SUPPORT SUPPORT SUPPORT

cathedral columns, old-fashioned underwear

when I get confused and frustrated

people willing to help when I'm lost in the woods

being there when down

being seen in a blizzard

SUPPORT SUPPORT SUPPORT SUPPORT

like being rescued, like what I get from you

meeting people in my situation, participation in many groups

accessing expert advice and information

helps me live my life independently, something I cannot give enough of

what makes me go on

S U P P O R T

The poet's explanation of his process

This poem uses elements of a type of poetry known as "concrete." Concrete poems use space in ways that other forms of poem do not. The words are not merely descriptions or narrative tools, but dramatize the underlying meaning of the poem by the way in which they appear on the page—size, arrangement, colour, typeface. They draw attention to themselves, becoming not only something to read but something to see.

One of the first things that struck me about this group of responses, aside from the strong, evocative images, was the repeating of the word "support." Seeing it eleven times made me think of bells chiming, each time a bit louder, and then it made me think of strong square bricks providing a foundation to a structure of images, metaphors, and emotions. The poetic lines are clustered, balanced, sometimes looking a bit fragile or likely to tip from the weight of words above them, but the underlying support structure is always there to hold them steady.

Source Material:

Support helps me live my life independently
Support gives me the confidence to live with dementia
Support groups shows me that I'm not alone
Support helps me when I get confused and frustrated
Support opens their ears, when I just want to talk

Support to me, is provided by my participation in many groups, also and most importantly by my wife, without whom I could not manage anywhere near a normal life, with PCA
Support is like being rescued, like being seen in a blizzard or knowing there's someone to catch you when the Tsunami hits. It's having people willing to help through action when you yourself feel helplessly lost in the woods.
Support means something I cannot give enough of. Nothing seems to help
Support means
enablement
kicking ass
being there when down
Support is the lighthouse in a storm
Support means caring
Something that makes me go on, like what I get from you
A listening ear
Meeting people in my situation
Accessing expert advice and information.
Old fashioned underwear
cathedral columns
The spine
Holding my husband up

7.3 ***What does support mean to you?*** [mixed rarer dementia group]

What Else Could I Try To Do

Writing/spelling, language, communication, definitions…

Take a break when you need one

Seek advice when you need it

Learn from other's experiences

Learn from others that have PPA how they are managing

Support means I can manage/operate better longer

Do the best I can for as long as I can

Do your best, but when you fail do not be hard on yourself

Support means not feeling utterly alone

I am not in this alone

It means having someone to help bear the unbearable

Sharing with others how to help me in the future

A good source for me to continue in my life

To link to the item / person that I need to speak to / use

Able to connect / talk with others that have PPA and I am able to help / support them

Appreciate being involved in any research to help finding new treatments / management for those with PPA

Helps me feel better about myself as I can help others

Support means

I am not in this alone

The poet's explanation of her process:

The form of this free verse poem is a weave of two different kinds of lines to suggest how to make a supportive environment out of various voices and ideas. The lines flow back and forth and answer each other to suggest how to create support by listening carefully to those trying to help, and to oneself.

Source Material:

Support means to me a good source for me to continue in my life.
I am not in this alone
Learn from other's experiences
Do your best, but when you fail do not be hard on yourself
Take a break when you need one
Seek advice when you need it

Support helps me to try to do the best I can for as long as I can eg. writing/spelling, language, communication, definitions.
I am in the process of getting a new ipad and a SPL and Occupational Therapist are taking lots !!!! of pictures that I send to them of areas in my condo, friends, relatives etc. etc. and they are setting it up on my new ipad to assist me in being able to link to the item / person that I need to speak to / use. That is supportive knowing that as I can't communicate well, I have an ipad set up with lots of items on it that I can select to help me. That support means I can manage/operate better longer.
If I am able to connect / talk with others that have PPA and I am able to help / support them -- that is very helpful for me. Also helpful to learn from others that have PPA how they are managing it -- what else could I try to do.
I really appreciate being involved in any research -- to help finding new treatments / management for those with PPA -- of course right away !!!! but for those, of course, in the future. That is very supportive for me -- helps me feel better about myself as I can contribute to -- help others.
Sharing with others how to help me in the future is very supportive to me
Support means not feeling utterly alone. It means having someone to help bear the unbearable.

8.3 ***What does support mean to you?*** [mixed rarer dementia group]

A Journey

as we venture
through a journey
of so many unknowns
in need of understanding
advice
back-up
relief
you actually care enough
to learn about
this most wretched of diseases
you are aboard my train
and will help in any way you can
trusted lifetime friendships
that one can always rely on
for a laugh
eternally grateful
hugs, many of them

with whom you can share experiences
experts who listen objectively
and share their expertise

you
you get it
someone to talk to
my loved one, my children

words, a journal in-waiting
for my kids to discover who helped out

this is about me
it's not about you
you are aboard my train

The poet's explanation of her process:

In these responses, the idea of a journey, of movement, suggested the flow of the free verse poem, which goes gently from one thought to another and gathers all the ideas together into the theme of support.

Source Material:

Help to someone in need of understanding
situations, problems.
Advice
Back-up
Someone to talk to
Relief
Trusted lifetime friendships that one
can always rely on for a laugh
New friendships living the same
story with one whom can share experiences
Subject matter experts who listen
objectively and share their expertise
as we venture thru a journey of
so many unknowns.
Hugs, many of them :)

support means you actually care enough to learn about this most wretched of diseases
support means you are aboard my train and will help in any way you can
support means you you get it - this is about me, my loved one, my children; it's not about you and your inability to cope with my loved one's FTD diagnosis and how it impacts YOU
Words to go along with question = eternally grateful; a journal in-waiting for my kids to discover who helped out

---

9.3 ***What does support mean to you?*** [FTD cohort group]

Acceptance, Relief, Managing

Acceptance of the dementia brain
Worldwide cohesion
Acknowledgement
Well-organised
Advice to help you understand
Valuing
Assistance
Understanding
Being listened to
Support is essentially support
Bonding with others
Subtle
Carer not isolated in their own cosmos
Strength
Close friend who knows when I'm struggling
Someone to listen
Comfort
Someone or something to lean on
Compassion
So individual
Enabling us to live life to the full
Safe
Encouragement
Relief
Forgetting problems
Reliability

Friends become strangers and strangers become friends
    Reassurance when needed
Guiding community
    Phone calls from children who live far away
Having time for me
    Peacefully
Help is there
    Passing without a bother
Helping hand
    Others in the world
Holding of hands across all cultures
    Opening up into a universe
Infrastructure
    No judgement
Insight into dementia world
    Navigating your way through
Kindness
    Meeting others who are in the same place as you
Living for the moment when we sing
    Many forms
Managing

Explanation of the poet's process:

When I read the heartfelt responses to this prompt, "*What does support mean to you?*" the first thing I noticed was the letter A in a number of places, plus a scattering of Bs and several Cs, and I immediately thought about an alphabet poem, where the opening words of each line are placed in alphabetical order. This, I thought, might provide a sturdy underlying structure to the topic of "support," and it has been my experience in the past that adhering to strict, almost mathematical rules of putting a poem together can work to strong effect. In this case, though, there wasn't a response for every letter of the alphabet, so the concept needed to be adapted.

As many of the responses were single words or short phrases, with others more extensive, the first thing I did was to break apart some of the longer expressions into separate images using words from all participants. I changed a few of the verb tenses and then alphabetised the list…and it didn't quite work. The simple structure of the alphabet didn't provide the proper support for a poem that needed to be all *about* the support.

And then I remembered the “mirror” poetic form that I’d encountered many years ago, where the poem can be read in a conventional manner from top to bottom, and then change direction and be read from bottom to top. When I combined this with the alphabet poem construction, I started seeing thought-provoking juxtapositions of the participants’ images and emotions. The title, “Acceptance, Relief, Managing,” derives from the first word, the word at the halfway point, and the final word.

Source Material:

Listening
Accepting
Valuing
Someone to listen
Re-assurance when needed
Comfort and encouragement
Being listened to
Reliability
Having time for me
Enabling us to live life to the full
An opening up into a universe
where others in the world
have insight into Dementia WORLD
Worldwide cohesion
Portuguese friend’s mother
Passed today without a bother
Peacefully
A holding of hands across all
CULTURES
In acceptance of the dementia brain
SO individual
CARER is not isolated in their own Cosmos
No judgement
Compassion and understanding
Support for me as I care for my husband with profound dementia comes in many forms....
the structured support from the wonderful infrastructure that has been developed in our welfare state ...yes I pay for it ...over £400 per month but it’s structured well organised and safe ...this support has enabled me to look after my husband at home
Support specifically for me is more subtle ......phone calls from children who live away...from sisters with their own problems who live close by ....from a new but close friend who knows when I’m struggling...from my Guiding community where I

still manage to be an assistant leader ....from a carers choir where I can forget problems and live for the moment when we sing
I have read ..."when someone gets dementia friends become strangers and strangers become friends " ....it's true
Relief
Acknowledgement that support is needed and help is there.
A helping hand
Someone or something to lean on
Kindness
Strength
Advise to help you understand and how to navigate your way through
Meeting others who are in the same place as you and who understand
Somewhere to form bonds with others
Support is essentially support.

10.3 ***What does support mean to you?*** [mixed rarer dementia group]

Help Me

It's a range of things --
compassion, understanding, love --
someone who listens … and believes me
someone who helps when I'm down and out
someone who reaches out
and gives me another path or crutch (an option)
someone to hug me and tell me it's ok
someone who can take over when it's too much

In between
there is support that helps me see things differently
and support that just takes time
to listen and see things the way I do,
not judged by expression of my thoughts or feelings

Never tell me I'm not doing enough for my husband --
just step in to help me!
Help me, so I can help him.
Honour his wishes.

Help is a range of things.
(an option: leave cookies on our porch!)

The poet's explanation of her process:

"Help Me" is an interplay of voices commenting very personally on the human condition of living with dementia. The poem speaks to the kinds of individuals who can be helpful, gives a framework for how they can help, and references a particular case that poignantly illustrates the theme.

Source Material:

Help me, so I can help him.
Leave cookies on our porch.
Honour his wishes.
compassion, understanding, love, not judged by expression of my thoughts or feelings
Someone who listens….and believes me
Someone who helps when I'm down and out.
Someone who reaches out and gives me another path or crutch (an option)
Never tell me I'm not doing enough for my husband; just step in to help me!
It's a range of things.
From someone to hug me and tell me it's ok
To someone who can take over when it's too much
In between there is support that helps me see things differently and support that just takes time to listen and see things the way I do

---

11.3 ***What does support mean to you?*** [Cohort group PPA/FTD]

To Continue

I get advice
truly interested
not feeling alone
I am not on my own

non-judgemental people
creation of PPA network
my friends do talk with me
I want to hear positive things

it lifts me up in times of doubt
giving me a degree of comfort
listen to what I am trying to say
it does well for me to be with them

taking time and emotion to understand me
allowing me to continue to do the things I enjoy
allowing me to still participate in decision making
being there for me to continue doing the things I can

enjoy, or at least not be frustrated, having to care for me
it allows me to move forward in a positive way with my life

The poet's explanation about his process:

In reading over the responses to this question, I was immediately struck by the contrast between the brief, concise lines and the more lyrical, sentence-like ones. Some of these longer lines I split into two pieces, and then, after changing a few verb tenses, arranged them all by length. The resulting poem places participants' responses in new configurations, highlighting the connections and contrasts between them. It also provides the visual impression of an architectural buttress, which reinforces the theme of support.

Source Material:

listens to what I am trying to say
Non-judgemental people
I am not on my own
I get advice
I want to hear positive things ...
my friends do talk with me and it does well for me to be with them.
It allows me to continue to do the things I enjoy.
It lifts me up in times of doubt.
It allows me to move forward in a positive way with my life.
Not feeling alone
Creation of PPA network
It gives me a degree of comfort
Truly interested and taking time and emotion to Understand me.
Enjoy, at least not be frustrated in having to care for me.

Allowing me to still participate in decision making and being there for me to continue doing things I can.

12.3 ***What does support mean to you?*** [mixed rarer dementia group]

The Resiliency

Support feels like being wrapped in an old comfy sweater,
the kind that I don't have to ask for it just shows up,
neighbours and friends asking if they can lend a hand,
family,
family being by your side.

Someone to listen to me without judgment,
workers willing to lend an ear,
unconditional actions to ease the burden of care,
companionship, less stress, stimulation,
acceptance, acknowledgment, understanding.

joy in every day

I know you think I'm crazy sometimes
and question my judgment
on every decision I make
but if you want to show me your support
learn to accept me as I am,
with all my faults and errors,
don't scold me like a child,
just cover for me and let me believe
in the resiliency found in love.

Explanation of the poet's process:
Responses in this group included what was clearly a finished poem that wrapped up the strands of the other thoughts and became the "old comfy sweater" in itself. The responses are the yarn that knit into the final lovely resilient garment.

Source Material:

Support me by showing acceptance, acknowledgment, understanding.
Support feels like being wrapped in an old comfy sweater
The best support is the kind that I don't have to ask for it just shows up
Support means to me:
Family
Unconditional actions to ease the burden of care
Someone to listen to me without judgement
I know you think I'm crazy sometimes
And question my judgement
On every decision I make

13.3 ***What does support mean to you?*** [mixed rarer dementia group]

Please Stick Around

Seeing me without judgment.

Hope that we can cope.

I feel less alone to know others understand

or have travelled this road as well.

Knowledge and strength from shared experience.

Simple yet flexible.

Encouragement.

Willingness not Reluctance

Optimism not Pessimism

Pro-action not Reaction

Action not Procrastination

I am so blessed to have such amazing supports in my life,

a friend who texts me everyday with a nice thought,

family members willing to help each other,

caring, helping with visits, hugs, drives,

and professional help to educate doctors and general public.

Walk with him and listen --

support needs will change,

taking time to listen and giving time for talking,

providing support strategies that are specific to my condition.

A genuine appreciation and thankfulness for the willing,

the kind and the pleasant;

understanding what to expect allows preparing for it,

so

please, stick around.

The poet's explanation of her process:

This free-verse poem does what it describes: it is "simple yet flexible", delivering "knowledge and strength from shared experience". The poem both listens and talks, and after asking in the title that the audience stick around, explores what kind of knowledge is needed to enable those willing to "stick around" to be helpful.

Source Material:

Everything.
I am so blessed to have such amazing supports in my life.
I feel less alone to know others understand or have travelled this road as well.
Knowledge and strength from shared experience
Encouragement
Hope that we can cope
Kindness
A friend who texts me everyday with a nice thought.
Seeing me without judgement.
please stick around
walk with him and listen
simple yet flexible
As we go through this together know that:
support needs will change

understanding what to expect allows preparing for it
being proactive is better than being reactive
family members need to be willing to help each other
Taking time to listen and giving time for talking
Providing support strategies that are specific to my condition
Willingness not Reluctance
Optimism not Pessimism
Proaction not Reaction
Action not Procrastination
A genuine Appreciation and Thankfulness for the Willing, the Kind and the Pleasant
Support for my FTD could be listening, believing, searching the illness online to help us.
Caring, helping with visits, hugs, drives and professional help to educate doctors and general public.
But if you want to show me your support
Learn to accept me as I am,
With all my faults and errors
Don't scold me like a child
Just cover for me and let me believe
In the resiliency found in love
Support is family being by your side
Support is neighbours and friends asking if they can lend a hand
Support is workers willing to lend a ear
To me support means less stress
Always include stimulation
Always include companionship
Always include joy in every day

End of data set

Thank you to the funders of this research:

UKRI Economic and Social Research Council
FUNDED BY NIHR | National Institute for Health and Care Research
